# Combination of the novel histone deacetylase inhibitor YCW1 and radiation induces autophagic cell death through the downregulation of BNIP3 in triple-negative breast cancer cells in vitro and in an orthotopic mouse model

**DOI:** 10.1186/s12943-016-0531-5

**Published:** 2016-06-10

**Authors:** Hui-Wen Chiu, Ya-Ling Yeh, Yi-Ching Wang, Wei-Jan Huang, Sheng-Yow Ho, Pinpin Lin, Ying-Jan Wang

**Affiliations:** Division of Nephrology, Department of Internal Medicine, Shuang Ho Hospital, Taipei Medical University, Taipei, Taiwan; Graduate Institute of Clinical Medicine, College of Medicine, Taipei Medical University, Taipei, Taiwan; Department of Environmental and Occupational Health, College of Medicine, National Cheng Kung University, 138 Sheng-Li Road, Tainan, Taiwan 704; Institute of Basic Medical Sciences, National Cheng Kung University, Tainan, Taiwan; Department of Pharmacology, National Cheng Kung University, Tainan, Taiwan; Graduate Institute of Pharmacognosy, Taipei Medical University, Taipei, Taiwan; Department of Radiation Oncology, Chi Mei Medical Center, Liouying, Tainan, Taiwan; Chang Jung Christian University, Tainan, Taiwan; National Institute of Environmental Health Sciences, National Health Research Institutes, No. 35 Keyan Road, Zhunan Town, Miaoli County 350 Taiwan; Department of Biomedical Informatics, Asia University, Taichung, Taiwan; Department of Medical Research, China Medical University Hospital, China Medical University, Taichung, Taiwan

**Keywords:** Histone deacetylase inhibitor, Radiation, Triple-negative breast cancer, Autophagy

## Abstract

**Background:**

Triple-negative breast cancer (TNBC) is the most aggressive and invasive of the breast cancer subtypes. TNBC is a challenging disease that lacks targets for treatment. Histone deacetylase inhibitors (HDACi) are a group of targeted anticancer agents that enhance radiosensitivity. Bcl-2/adenovirus E1B 19 kDa protein-interacting protein 3 (BNIP3) is a member of the Bcl-2 subfamily. BNIP3 is not found in normal breast tissue but is up-regulated in breast cancer. In the present study, we investigated the anti-cancer effects of a newly developed HDACi, YCW1, combined with ionizing radiation (IR) in TNBC in vitro and in an orthotopic mouse model. Furthermore, we examined the relationship between autophagy and BNIP3.

**Methods:**

Trypan blue exclusion was used to investigate the viability of 4 T1 (a mouse TNBC cell line) and MDA-MB-231 cells (a human TNBC cell line) following combined YCW1 and IR treatment. Flow cytometry was used to determine apoptosis and autophagy. The expression levels of BNIP3, endoplasmic reticulum (ER) stress- and autophagic-related proteins were measured using western blot analysis. An orthotopic mouse model was used to investigate the in vivo effects of YCW1 and IR alone and in combination. Tumor volumes were monitored using a bioluminescence-based IVIS Imaging System 200.

**Results:**

We found that YCW1 significantly enhanced toxicity in 4 T1 cells compared with suberoylanilide hydroxamic acid (SAHA), which was the first HDACi approved by the Food and Drug Administration for clinical use in cancer patients. The combined treatment of YCW1 and IR enhanced cytotoxicity by inducing ER stress and increasing autophagy induction. Additionally, the combined treatment caused autophagic flux and autophagic cell death. Furthermore, the expression level of BNIP3 was significantly decreased in cells following combined treatment. The downregulation of BNIP3 led to a significant increase in autophagy and cytotoxicity. The combined anti-tumor effects of YCW1 and IR were also observed in an orthotopic mouse model; combination therapy resulted in a significant increase in autophagy and decreased tumor tissue expression of BNIP3 in the tumor tissue.

**Conclusions:**

These data support the possibility of using a combination of HDACi and IR in the treatment of TNBC. Moreover, BNIP3 may be a potential target protein for TNBC treatment.

**Electronic supplementary material:**

The online version of this article (doi:10.1186/s12943-016-0531-5) contains supplementary material, which is available to authorized users.

## Background

Triple-negative breast cancer (TNBC) characterized by the absence of estrogen receptor alpha (ER), progesterone receptor (PR) and human epidermal growth factor receptor 2 (HER2) expression, is a basal-like subgroup of breast cancers that accounts for 10–20 % of all breast cancers [1]. Patients with this subtype are more likely to develop recurrence within the first 5 years, and survival following metastatic relapse is shorter for TNBC patients than those with other breast cancer subtypes [2]. Currently, TNBC is one of the most attractive areas in cancer research. One reason for this scientific interest is the lack of therapeutic targets for TNBC. Therefore, identifying biological markers of TNBC progression could be helpful for the prevention of breast cancer metastasis and could provide novel therapeutic strategies for the disease. TNBC is typically treated with surgery, radiotherapy, and chemotherapy. Overcoming the deleterious consequences of radiotherapy and maximizing its anti-tumor effects to control tumor progression should be the goal of combined radio- and chemotherapy. Combination therapies aim to enhance radiosensitivity and prevent tumor recurrence. Numerous conventional cytotoxic drugs are used in conjunction with different radiation techniques [3]. Recently, data accumulated by us and others have revealed that some compounds or drugs enhanced radiosensitivity through regulation of the cell cycle, induction of cell death and inhibition of DNA repair [1, 4–6].

Ionizing radiation (IR) induces important signal transduction pathways, such as the PI3K pathway, that are linked with radioprotective and growth-promoting events [7]. The PI3K signaling pathway is associated with major radioresistance mechanisms, such as intrinsic radiosensitivity, tumor cell proliferation and hypoxia [8]. Downstream molecular targets of PI3K up-regulate hypoxia-related proteins, stimulate mitogenic and pro-survival pathways and have anti-apoptotic effects via the induction of Bcl-XL, which is a member of the Bcl-2 family, and the inactivation of Bad and procaspase-9 [9]. Positive Bcl-2 expression has been associated with poor survival and reduced sensitivity to chemotherapy in patients with TNBC [10]. Bcl-2/adenovirus E1B 19 kDa protein-interacting protein 3 (BNIP3) is a member of the Bcl-2 subfamily of death-inducing mitochondrial proteins [11]. Previous studies have demonstrated that BNIP3 provides a survival advantage in cancer cells by promoting autophagy and eliminating damaged mitochondria with low membrane potential that are a source of intracellular ROS [12, 13]. Additionally, BNIP3 expression is restricted to few normal tissues, including skeletal muscle and brain [14]. In contrast to normal breast tissue in which BNIP3 was not expressed up-regulation of BNIP3 was observed in breast cancer [15]. However, whether BNIP3 has an important role in TBNC remains unknown.

Many studies have implicated that HDAC enzymes have a role in the development of cancer and, therefore, are potential therapeutic targets [16, 17]. HDAC inhibitors (HDACi) block the deacetylation function of HDACs, causing cell cycle arrest, endoplasmic reticulum (ER) stress, differentiation, inhibition of angiogenesis, apoptosis and autophagy in many tumors [6, 17]. Normal cells are relatively resistant to HDACi-induced cell death [18]. Moreover, HDACi can affect apoptosis and autophagy through regulation of the Bcl-2 family including inhibition of Bcl-2 and activation of Bax [19]. However, several serious adverse events were reported in patients treated with suberoylanilide hydroxamic acid (SAHA), which was the first HDACi approved by the US Food and Drug Administration (FDA) for the clinical treatment of cutaneous T-cell lymphoma, and other HDACi [20]. Therefore, the development of novel HDACi combination therapies or new HDACi with improved efficacies is urgently needed, particularly for solid tumors.

In this study, we used a newly developed HDACi, YCW1 (octanedioic acid [3-(2-(5-methoxy-1H-indol-1-yl)ethoxy)phenyl]-amide N-hydroxyamide), which was been optimized for HDAC inhibition using structure-based analyses [21, 22]. The murine TNBC cell line 4 T1 and human TNBC cell line MDA-MB-231 were used to investigate the anti-tumor effects of IR combined with this novel HDACi (YCW1) and the underlying mechanism of these effects, including the types of cell death and ER stress. Furthermore, we tested the inhibitory effect of IR combined with YCW1 in a 4 T1 orthotopic breast cancer model in mice. Our results suggest that the downregulation of BNIP3 in TNBC cells significantly increased the anti-tumor effects of IR and YCW1 through the induction of autophagic cell death. Using an orthotopic breast cancer mouse model of TNBC cells, we verified that co-treatment with IR and YCW1 inhibited tumor growth.

## Results

### YCW1 inhibits HDAC activity and enhances cytotoxicity of IR in TNBC cells

**Fig. 1:**
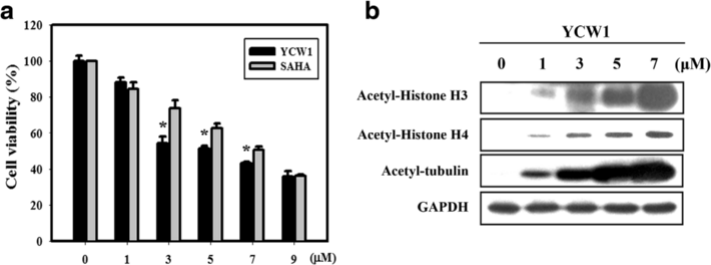
Cytotoxic effects resulting from SAHA and YCW1 treatment in 4 T1 cells. **a** Dose-dependent effects of SAHA and YCW1 treatment on the viability of 4 T1 cells for 24 h. *, *p* < 0.05, SAHA versus YCW1. Data are presented as the mean ± standard deviation of three independent experiments. **b** Concentration-dependent effects of YCW1 on histone and nonhistone proteins for 24 h in 4 T1 cells

**Fig. 2:**
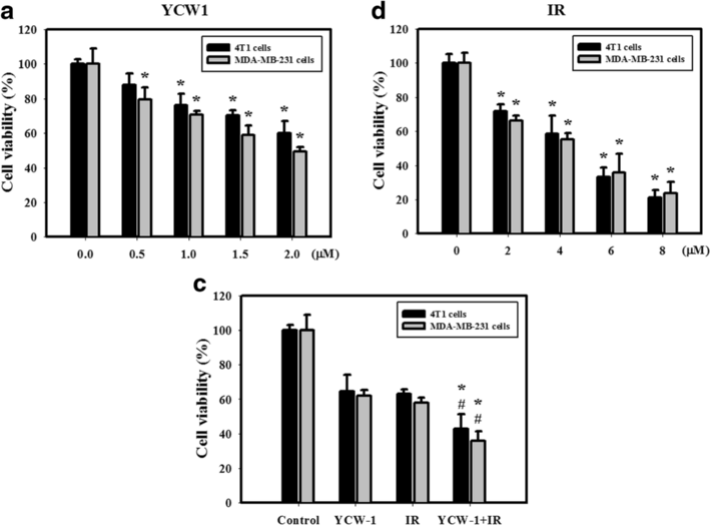
Cytotoxic effects resulting from YCW1 and/or IR in 4 T1 and MBA-MD-231 cells. **a** Concentration-dependent effects of YCW1 on the viability of 4 T1 and MBA-MD-231 cells. Cells were treated with 0.5, 1, 1.5 or 2 μM of YCW1 for 48 h. **b** Dose-dependent effects of IR on the viability of 4 T1 and MBA-MD-231 cells. Cells were treated with 2, 4, 6 or 8 Gy of IR for 48 h. *, *p* < 0.05, YCW1 or IR versus control group. **c** Cytotoxicity observed in cells treated with YCW1 (1 μM) and IR (4 Gy) for 48 h in 4 T1 and MBA-MD-231 cells. #, *p* < 0.05, IR versus combined treatment. *, *p* < 0.05, YCW1 versus combined treatment. Data are presented as the mean ± standard deviation of three independent experiments

SAHA was the first HDACi to be approved by the FDA for clinical use in cancer patients. However, some serious adverse events were reported in patients treated with SAHA [23]. Therefore, we investigated the use of a novel HDACi (YCW1). Our previous study indicated that YCW1 is a class I and II HDACi. Furthermore, compared with SAHA, YCW1 showed better inhibition of total HDAC activity [22]. In the present study, we found that YCW1 significantly enhanced toxicity in 4 T1 cells compared with SAHA in 4 T1 cells (Fig. 1a). To further determine whether YCW1 inhibits HDAC activity in 4 T1 cells, the murine TNBC cell line was treated with YCW1 at the indicated doses to examine the acetylation level of HDAC target proteins. YCW1 induced dose-dependent acetylation of histones H3 and H4, and non-histone protein tubulin (Fig. 1b). Next, we investigated the effects of YCW1 concentration and IR dose on 4 T1 and MDA-MB-231 cell viability (Figs. 2a and b). YCW1 and IR treatment both decreased cell viability in a concentration- and dose-dependent manner, respectively. As shown in Fig. 2c, the combined treatment with YCW1 and IR enhanced the growth inhibition of 4 T1 and MDA-MB-231 cells compared with YCW1 or IR alone.

### Combined treatment induces ER stress and autophagy in 4 T1 cells

**Fig. 3:**
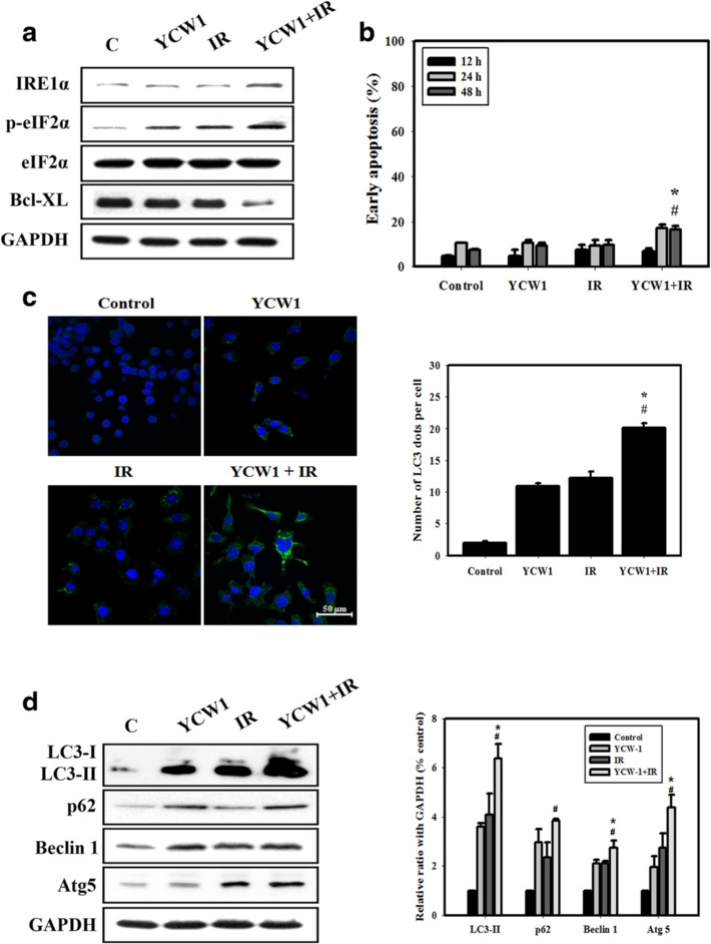
Measurement of cell death and ER stress in 4 T1 cells. **a** The expression levels of ER stress-related proteins were measured by western blot analysis following treatment with YCW1 and IR alone or in combination. Cells were treated with YCW1 (1 μM) and IR (4 Gy) for 12 h. **b** Early apoptosis, detected using an Annexin V apoptosis detection kit, was measured using flow cytometry. Cells were treated with YCW1 (1 μM) and IR (4 Gy) for 48 h. **c** Immunofluorescence staining of the LC3 protein in 4 T1 cells treated with YCW1 (1 μM) and IR (4 Gy) for 48 h. Representative cell images showing punctate LC3 distribution using a confocal microscope. Quantitative data calculating the number of LC3 dots per cell. #, *p* < 0.05, IR versus combined treatment. *, *p* < 0.05, YCW1 versus combined treatment. Data are presented as the mean ± standard deviation of three independent experiments. **d** The expression levels of autophagic-related proteins were measured by western blot analysis following treatment with YCW1 and IR alone or in combination. The densities of the bands were quantified with a computer densitometer (AlphaImager™ 2200 System Alpha Innotech Corporation, CA, USA). Cells were treated with YCW1 (1 μM) and IR (4 Gy) for 48 h. #, *p* < 0.05, IR versus combined treatment. *, *p* < 0.05, YCW1 versus combined treatment

**Fig. 4:**
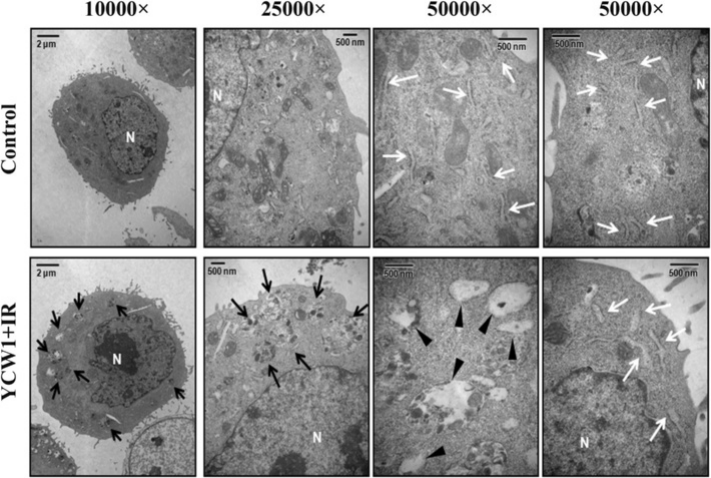
The ultrastructures of 4 T1 cells treated with YCW1 (1 μM) and IR (4 Gy) for 48 h were analyzed by TEM. N, nucleus. Black arrows, autophagosomes. Black arrowheads, autolysosome. White arrows, endoplasmic reticulum

Previous studies have demonstrated that HDACi and IR induce ER stress [24, 25]. Therefore, we determined whether combined treatment with YCW1 and IR induced an ER stress response in 4 T1 cells. Our results showed that levels of inositol-requiring enzyme 1α (IRE1α) and eukaryotic initiation factor 2α (eIF2α) phosphorylation, both markers of ER stress, increased in cells treated with combined YCW1 and IR compared with those subjected to individual treatment (Fig. 3a). Members of the Bcl-2 family act as sentinels that selectively trigger apoptosis in response to developmental cues or stress-signals such as ER stress [26]. We found that the Bcl-XL protein expression levels decreased in 4 T1 cells following combined treatment. Furthermore, we analyzed the types of cell death induced by combined treatment. Our results showed that the occurrence of early apoptosis in 4 T1 cells treated with YCW1 and/or IR was low (Fig. 3b). We further analyzed the occurrence of type II programmed cell death, autophagy. Microtubule-associated protein light chain 3 (LC3) is widely used to monitor autophagy [27]. Thus, we applied fluorescence microscopy to determine the percentage of cells with punctate LC3 staining (Fig. 3c). Quantitative results showed a significant increase in LC3 immunopositive dots in 4 T1 cells that received the combined treatment compared with YCW1 or IR alone. The expression of autophagic-related LC3-II, beclin 1, p62 and Atg5 proteins in 4 T1 cells after different treatments were analyzed (Fig. 3d). We found a marked increase in the expression of LC3-II, beclin 1, p62 and Atg5 proteins in 4 T1 cells treated with YCW1 and IR, alone and in combination. The combined treatment showed a significant increase in the expression of LC3-II, beclin 1 and Atg5 proteins compared with YCW1 or IR treatment alone. By TEM, 4 T1 cells exhibited obvious autophagic vacuoles in the cytoplasm after combined treatment (Fig. 4 and Additional file 1: Figure S1B). Additionally, combined treatment caused a severe dilation of ER membranes, which is a sign of ER stress, compared with the control group. These results confirm that the combined treatment induced ER stress and autophagy in 4 T1 cells.

### Autophagic cell death is induced by the combination of YCW1 and IR

**Fig. 5:**
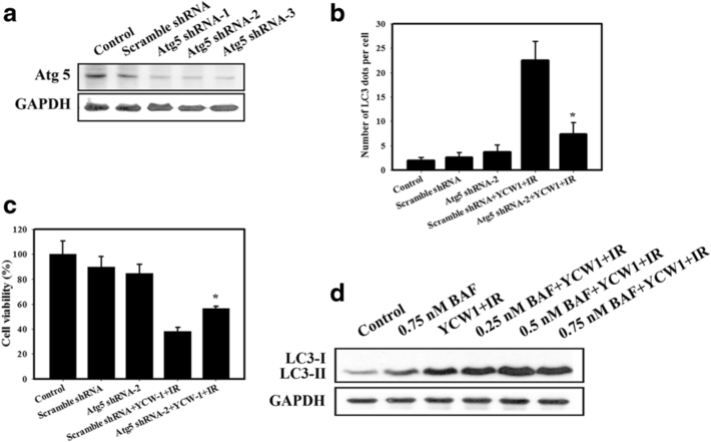
Measurement of autophagic and cytotoxic effects in 4 T1 cells pretreated with Atg shRNA. **a** Western blotting for Atg5. The cells were transfected with Atg5 shRNA for 24 h. **b** Quantitative data calculating the number of LC3 dots per cell in the absence or presence of Atg5 shRNA. **c** Cytotoxic effects in the absence or presence of Atg5 shRNA. Cells were transfected with Atg5 shRNA for 24 h and were then incubated with YCW1 (1 μM) and IR (4 Gy) for 48 h. *, *p* < 0.05, YCW1 + IR versus Atg5 shRNA + YCW1 + IR. Data are presented as the mean ± standard deviation of three independent experiments. **d** Western blot analysis of LC3-I and LC3-II expression in 4 T1 cells. Cells were pretreated with BAF for 1 h prior to IR treatment and then treated with YCW1 (1 μM) and IR (4 Gy) for 48 h

Autophagy is a cytoprotective process in starving cells. However, excess autophagy can induce type II programmed cell death (autophagic cell death) [28]. To study the role of autophagy in combined treatment-induced cytotoxicity, cells were transfected with Atg5 shRNA then exposed to YCW1and IR. Atg5 shRNA inhibited the expression of Atg5, LC3 puncta and autophagic vacuoles (Fig. 5a, b and Additional file 1: Figure S1). Furthermore, inhibition of autophagy by Atg5 shRNA increased the cell viability in 4 T1 cells treated with the combined treatment (Fig. 5c). Previous studies have suggested that one of the criteria of autophagic cell death is an increase in autophagic flux, and not just an increase in the autophagic markers [29]. We analyzed the autophagic flux with bafilomycin A1(BAF, a vacuolar-type H^+^-ATPase inhibitor that blocks autophagosome-lysosome fusion). As shown in Fig. 5c, combined treatment led to the accumulation of LC3-II in the presence of BAF. Therefore, the increase in LC3-II was not due to the blockade of autophagic degradation. The increase of LC3-II by BAF in cells treated with combined treatment likely results in autophagic flux.

### BNIP3 expression influences the cytotoxic effects of YCW1 and IR in 4 T1 cells

**Fig. 6:**
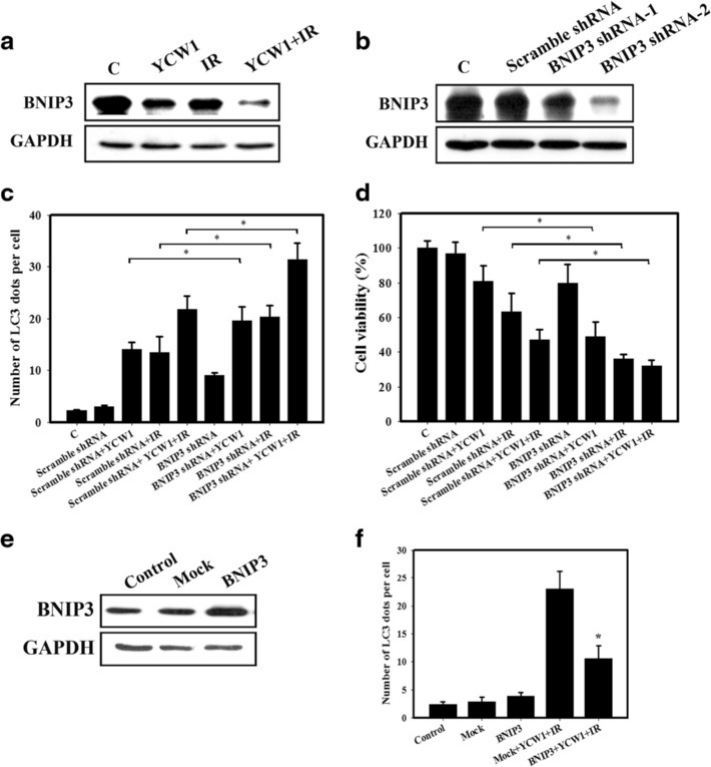
BNIP3 knockdown by shRNA abrogates the growth of 4 T1 cells. **a** The expression levels of BNIP3 in 4 T1 cells were analyzed by western blot analysis following YCW and/or IR treatment. Cells were treated with YCW1 (1 μM) and IR (4 Gy) for 48 h. **b** Transfection efficacy was verified by western blot analysis. BNIP3 protein expression in 4 T1 cells transfected with scramble or BNIP3 shRNA for 24 h. **c** Quantitative data calculating the number of LC3 dots per cell transfected with scramble or BNIP3 shRNA. *, *p* < 0.05. **d** Cytotoxicity observed in cells transfected with scramble or BNIP3 shRNA using flow cytometry. *, *p* < 0.05. **e** Western blot analysis of BNIP3 protein expression in cells overexpressing BNIP3. **f** Quantitative data calculating the number of LC3 dots in the overexpression of BNIP3. Cells were transfected with BNIP3 for 24 h and were then incubated with YCW1 (1 μM) and IR (4 Gy) for 48 h. *, *p* < 0.05, YCW1 + IR versus BNIP3 + YCW1 + IR. Data are presented as the mean ± standard deviation of three independent experiments

To investigate whether BNIP3 is involved in combined treatment-induced cell death, we performed western blotting to detect its protein expression levels. The results showed that BNIP3 levels were significantly decreased in cells treated with combined treatment compared with those treated with YCW1 and IR alone (Fig. 6a). Next, BNIP3 protein expression was depleted using shRNA (Fig. 6b). We found that BNIP3 shRNA-2 inhibited more BNIP3 expression compare with BNIP3 shRNA-1. Thus, we used BNIP3 shRNA-2 to downregulate BNIP3 and investigated the role of BNIP3 in combined treatment-induced cytotoxicity and autophagy. We observed that a significant increase in LC3 immunopositive dots was observed in 4 T1 cells transfected with BNIP3 shRNA compared with the control shRNA (Fig. 6c). Additionally, BNIP3 shRNA reduced the viability of 4 T1 cells (Fig. 6d). To further confirm the role of BNIP3 in autophagy activation, autophagy activity was measured in 4 T1 cells expressing BNIP3. After transfection, BNIP3 expression was significantly increased (Fig. 6e). We found that the LC3-puncta formation showed decreased when BNIP3 is overexpressed (Fig. 6f).

### Tumor growth in an orthotopic breast cancer model is suppressed by combined treatment with YCW1 and IR through the induction of autophagy and the inhibition of BNIP3

**Fig. 7:**
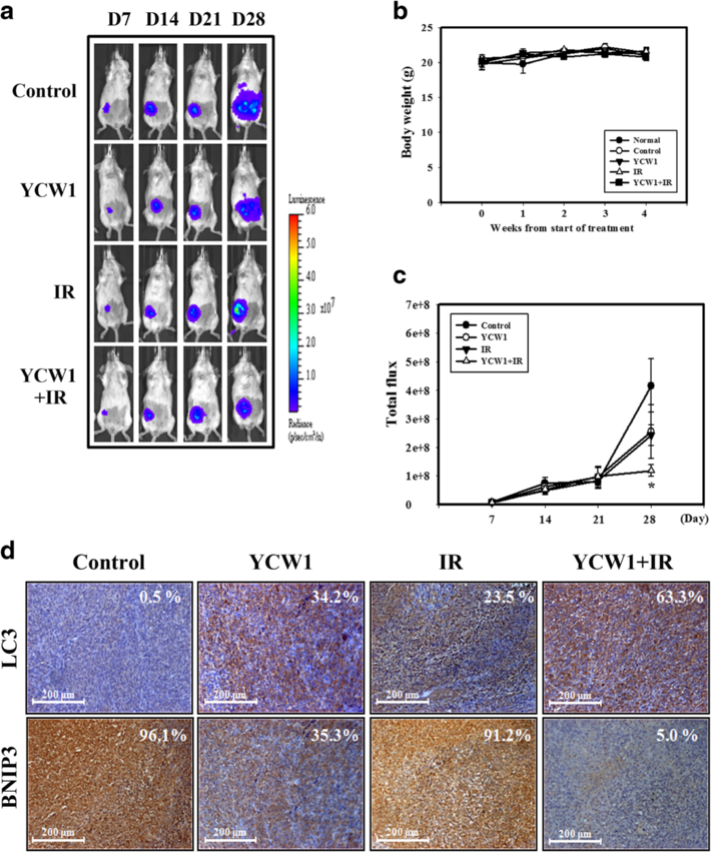
Combined treatment with YCW1 and IR enhances the anti-tumor effects in an orthotopic breast cancer model. **a** 4 T1-luc cells were injected into the mammary fat pads of Balb/c mice, observed for luciferase signals and photographed using an IVIS 200. **b** Measurement of body weight in Balb/c mice taken once per week. **c** Quantification of the luciferase signals. *, *p* < 0.05, versus control. **d** IHC staining of orthotopic tumor tissues from the mice. IHC was used to determine the expression levels of LC3 and BNIP3 (×100 objective magnification). The percentage of LC3 and BNIP3-positive cells was determined using HistoQuest software (TissueGnostics)

We next evaluated the anti-tumor growth effect of YCW1 and IR alone, or in combination in vivo. Tumors were induced by the injection of 4 T1-Luc cells into the lactiferous ducts of the 4th mammary fat pads of female Balb/c mice. We measured the body weight and tumor volume of the mice every week. None of the treatment regimens produced any obvious signs of toxicity based on the loss of body weight (Fig. 7b). Furthermore, no detectable toxicity was evident by biochemical examination following treatment with YCW1 or IR alone (Table 1). In addition, we used an IVIS system to analyze bioluminescence. The results showed a gradual increase in tumor volume in the control group in a time-dependent manner. The combined treatment suppressed tumor volume compared with the control group (Fig. 7a and c). Next, the expression patterns of LC3 and BNIP3 in the 4 T1 tumors were examined by IHC staining (Fig. 7d). BNIP3 expression was decreased in the YCW1 alone, IR alone and combination groups compared with the control group. By contrast, the expression level of LC3 was increased. Tumor tissues from the mice treated with both YCW1 and IR showed lower BNIP3 and higher LC3 expression compared with the tumor tissues from mice treated with a single agent. This experiment showed that the combination of YCW1 and IR significantly inhibited BNIP3 and induced autophagic cell death in an orthotopic breast cancer model.

**Table 1:** Biochemical tests including GOT, GPT, albumin and BUN

		4 T1-Luc cells			
Item/Group	Normal	Control	YCW1	IR	YCW1 + IR
GOT (U/l)	137.67 ± 42.17	147.33 ± 13.72	184.00 ± 32.32	181.67 ± 29.35	180.67 ± 18.22
GPT (U/l)	48.00 ± 10.60	35.33 ± 3.18	33.67 ± 2.67	33.33 ± 2.19	40.33 ± 7.97
Albumin (g/dl)	2.67 ± 0.13	2.37 ± 0.24	2.37 ± 0.12	2.37 ± 0.15	2.23 ± 0.13
BUN (mg/dl)	21.60 ± 2.63	30.50 ± 10.42	26.73 ± 1.07	25.47 ± 1.60	26.53 ± 1.87

## Discussion

TNBC is the most invasive and aggressive among the breast cancer subtypes and there is no clinical therapy specific for patients with TNBC [30]. In the present study, we found that the combination of a novel HDACi (YCW1) and IR may have anti-tumor potential against TNBC both in vitro and in vivo (Figs. 2 and 7). Moreover, our previous study demonstrated that YCW1 induced a broad spectrum of anticancer activities in lung cancer cell and animal models. It has been showed that HDACi induced cell death in many tumors and had less toxicity in normal cells [17, 18]. However, HDACi have demonstrated limited clinical benefit for patients with solid tumors [31]. In our study, we used a novel HDACi, YCW1, and examined the combination effect of YCW1 and IR in TNBC cells. YCW1 showed a better inhibition of total HDAC activity and significantly enhanced toxicity compared with SAHA [22] (Fig. 1a). The combined treatment with YCW1 and IR enhanced the growth inhibition of 4 T1 and MDA-MB-231 cells compared with YCW1 or IR alone (Fig. 2). Furthermore, YCW1 caused no detectable toxicity as determined by either biochemical examination or in terms of the loss of body weight (Fig. 7b and Table 1). Therefore, YCW1 is a potential HDAC inhibitor and enhances radiosensitivity in TNBC cells.

HDACi cause ER stress, apoptosis and autophagy in many tumors [6, 17]. Previous research has shown that treatment of cancer cells with HDACi induced autophagy which promotes cancer cell survival when apoptosis induction is inhibited. Moreover, autophagy inhibition resulted in a higher level of apoptosis in response to SAHA treatment [32]. Rao et al. indicated that combination of autophagy inhibitor and HDACi induced the accumulation of toxic polyubiquitylated proteins and caused inhibitory effects on TNBC cell growth [33]. However, there are also numerous reports in the literature showing the pro-death function of autophagy. HDACi can induce caspase-independent autophagic cell death and have clear clinical implications in treating cancers with apoptotic defects [34, 35]. Therefore, the role of autophagy in regulating cancer cell death or survival remains controversial. Recent studies have investigated the induction of ER stress as a novel strategy for treating malignancies [36, 37]. ER stress triggers unfolded protein response (UPR) pathways, including the IRE1 pathway, the PKR-like ER-resistant kinase pathway and the activating transcription factor 6 pathway [38]. Data accumulated by us and others have revealed that IR activates ER stress and UPR pathways through the induction of DNA damage [24, 39]. Here, IRE1α and phosphorylated eIF2α, which are both UPR-related proteins, increased in cells treated with combined YCW1 and IR (Fig. 3a). Using TEM, the ultrastructures of the 4 T1 cells indicated ER stress after the treatment with combined treatment (Fig. 4). Evidence indicating that ER stress can induce cell death, including apoptosis and autophagy, has been reported [40, 41]. We found that combined treatment mainly induced autophagy and a small amount of apoptosis in 4 T1 cells (Figs. 3, 4 and Additional file 1: Figure S1). In our in vivo study, tumor tissues from the mice treated with combined treatment showed higher autopahgic levels compared with mice treated with a single agent (Fig. 7d). However, the role of autophagy in regulating cancer cell death or survival remains controversial. Furthermore, an increase in autophagic flux may be the a key factor that modulates autophagy to cell death [29]. The present study shows that Atg5 shRNA decreased combined treatment-induced cytotoxicity (Fig. 5c). Additionally, the combined treatment caused autophagic flux (Fig. 5d). Therefore, our results showed that the combination treatment with YCW1 and IR induced autophagic cell death in TNBC cells.

Members of the Bcl-2 family are contained in multiprotein complexes at the ER, where they regulate diverse cellular processes including autophagy, calcium homeostasis and the unfolded-protein response [42]. The Bcl-2 family comprises three subfamilies, an anti-apoptotic family, a pro-apoptotic multi-domain family and a pro-apoptotic BH3-only protein family [43]. Recent evidence shows that this family affects not only apoptosis but also autophagy [19, 44]. In the present study, Bcl-XL protein expression decreased in 4 T1 cells following combined treatment (Fig. 3a). Although BNIP3 is in the pro-apoptotic BH3-only protein family, it was recently found to increase resistance to apoptosis, and it was implicated that BNIP3 may also play an important role in autophagy [45, 46]. Moreover, growing evidence has shown that the overexpression of BNIP3 can be detected in many malignancies, such as glioma, breast cancer and prostate cancer [14, 47]. In addition, BNIP3 was not identified in normal breast but was up-regulated in breast cancer [15]. Park et al. indicated that BNIP3 degradation was triggered by autophagy and could be regulated by mTORC1 and AMPK [48]. In this study, we found that BNIP3 significantly decreased in cells or tumors following combined treatment compared with those that received YCW1 or IR alone (Figs. 6a and 7d). 4 T1 cells transfected with BNIP3 shRNA showed a significant increase the number of autophagic cells and reduced the viability compared with the control shRNA (Fig. 6c and d). Furthermore, the overexpression of BNIP3 decreased autophagy (Fig. 6f). Therefore, combined treatment induced autophagic cell death through the inhibition of BNIP3 in TNBC cells.

## Conclusions

Taken together, these data suggest that the combination of YCW1 and IR exerts a cytotoxic effect on TNBC cells that is associated with increased levels of autophagy and ER stress. More importantly, BNIP3 was degraded by the combined treatment-induced autophagy. This combination benefit was also observed in vivo using an orthotopic breast cancer model. Our data show that combining YCW1 and IR and targeting BNIP3 are therapeutic options that should be further investigated for the treatment of TNBC.

## Methods

### Preparation of YCW1

The complete chemical name of YCW1 is octanedioic acid [3-(2-(5-methoxy-1H-indol-1-yl)ethoxy)phenyl]-amide N-hydroxyamide. Requests for this compound should be sent to wjhuang@tmu.edu.tw.

### Cell culture

The murine breast cancer cell line 4 T1 (ATCC CRL-2539) and human breast cancer cell line MDA-MB-231 (ATCC HTB-26) were obtained from the American Type Culture Collection (ATCC). The luciferase-expressing murine breast cancer cell line 4 T1-Luc was obtained from Dr. M.L. Kuo (Institute of Toxicology, National Taiwan University, Taipei, Taiwan) [49]. The cells were cultured in Dulbecco’s modified essential medium (DMEM) (Gibco BRL, Grand Island, NY, USA) supplemented with an antibiotic containing 100 U/ml penicillin and 100 μg/ml streptomycin (Gibco BRL, Grand Island, NY, USA) and 10 % fetal bovine serum (HyClone, South Logan, UT, USA). The cells were incubated in a humidified atmosphere containing 5 % CO_2_ at 37 °C. Exponentially growing cells were detached using 0.05 % trypsin-EDTA (Gibco BRL, Grand Island, NY) in DMEM.

### Irradiation treatment and cell viability assay

IR was performed with 6 MV X-rays using a linear accelerator (Digital M Mevatron Accelerator, Siemens Medical Systems, CA, USA) at a dose rate of 5 Gy/min. An additional 2 cm of a tissue-equivalent bolus was placed on the top of the plastic tissue-culture flasks to ensure electronic equilibrium, and 10 cm of a tissue-equivalent material was placed under the flasks to achieve full backscatter. Cells were immediately treated with YCW1 following IR treatment. Next, the cells were centrifuged and resuspended in 0.1 ml PBS. Each cell suspension (0.02 ml) was mixed with 0.02 ml of a trypan blue solution (0.2 % in PBS). After 1 or 2 min, each solution was analyzed on a hemocytometer, with blue-stained cells counted as nonviable.

### Determination of early apoptosis

Apoptosis was assessed by quantifying the translocation of phosphatidylserine to the cell surface, detected with Annexin V staining (Calbiochem, San Diego, CA, USA). Experiments were conducted according to our previous reports [5, 50].

### Immunofluorescence microscopy

The cells were cultured on coverslips. Cells were harvested and fixed in 4 % paraformaldehyde and blocked with 1 % BSA for 30 min. This was followed by incubation with a specific antibody against LC3 (MBL, Japan) for 1 h. After washing, the cells were labeled with a DyLight™ 488-conjugated affinipure goat anti-rabbit IgG (Jackson Immuno-Research Laboratories, PA, USA) for 1 h and with DAPI. Finally, the cells were washed in PBS, covered with a coverslip, and examined with a fluorescence microscope or confocal microscope (Carl Zeiess LSM780, Instrument Development Center, NCKU). To quantify the number of LC3 dots per cell, a minimum of 50 cells per sample was counted.

### Transmission electron microscopy (TEM)

Cells were trypsinized and harvested, then fixed for 1 h in a solution containing 2.5 % glutaraldehyde and 2 % paraformaldehyde in 0.1 M cacodylate buffer, pH 7.3. After fixation, the samples were postfixed with buffer containing 1 % OsO_4_ for 30 min. Ultra-thin sections were subsequently observed under a transmission electron microscope (JEOL JEM-1200EX, Japan) at 100 kV.

### Western blot analysis

Total cellular protein lysate was prepared by harvesting cells in protein extraction buffer for 1 h at 4 °C, as described previously [4]. GAPDH expression represented the protein loading control. Anti-GAPDH, anti-BNIP3, anti-IRE1α, phospho-eIF2α and anti-beclin 1 antibodies were obtained from Abcam (Cambridge, MA, USA); anti-LC3 and anti-eIF2α antibodies were obtained from Abgent (San Diego, CA, USA); anti-acetyl-histone H3 and acetyl-histone H4 antibodies were obtained from Millipore (Bedford, MA, USA); anti-acetyl-tubulin antibody was obtained from Sigma (St. Louis, Missouri, USA); anti-Bcl-XL antibody was obtained from Cell Signaling Technology (Ipswich, MA, USA); and anti-p62/SQSTM1 antibody was obtained from MBL (Nagoya, Japan).

### Transfection of shRNA and plasmids

We used Arrest-In Transfection Reagent (Thermo, MA, USA) to transfect cells according to the manufacturer’s protocol. RNAi reagents were obtained from the National RNAi Core Facility located at the Institute of Molecular Biology/Genomic Research Center, Academia Sinica, supported by the National Core Facility Program for Biotechnology Grants of NSC (NSC 100-2319-B-001-002). The mouse library is referred to as TRC-Mm 1.0. Individual clones are identified as shRNA TRCN0000072184, shRNA TRCN0000009691, TRCN0000099432, TRCN0000099433, TRCN0000375819 and shRNA TRCN0000229458. BNIP3 plasmid was obtained from OriGene Technologies Inc. (Rockville, MD, USA).

### Orthotopic breast cancer model

All experiments on mice were performed according to the guidelines of our institute (the Guide for Care and Use of Laboratory Animals, Medical College, National Cheng Kung University). The animal use protocol listed below has been reviewed and approved by the Institutional Animal Care and Use Committee of National Cheng Kung University, Taiwan (Approval No: 104232). Six week-old female Balb/c mice were acquired from the National Laboratory Animal Center (Taiwan). The animals were housed five per cage at 24 ± 2 °C and 50 % ± 10 % relative humidity and subjected to a 12-h light/12-h dark cycle. 4 T1-Lluc cells (5 × 10^4^ cells in 0.2 ml of PBS) were injected into the lactiferous ducts of the 4th mammary fat pads in the female Balb/c mice. The mice were randomized into four treatment groups (5 mice per group): (1) Control (DMSO), (2) 25 mg/kg YCW1 three times per week for three weeks, (3) a single dose of 4 Gy IR, or (4) a combination of treatments with 25 mg/kg YCW1 three times per week and a single dose of 4 Gy IR. Bioluminescence imaging was conducted using an IVIS 200 imaging system coupled to a data acquisition computer running Living Image Software (XENOGEN). Before imaging, the mice were anesthetized with isoflurane and injected i.p with 150 mg/kg body weight endotoxin-free luciferase substrate (VivoGlo™, Promega). Body weights were measured once per week and used as an indicator of the systemic toxicity of the treatment. There were no deaths in any group during the experimental period. Mice were sacrificed via CO_2_ exposure. After sacrificing, the tumor tissues were formalin fixed and paraffin embedded for immunohistochemistry.

### Immunohistochemical (IHC) staining analysis

Paraffin-embedded tissue sections (4 μm) were dried, deparaffinized, and rehydrated. Following microwave pretreatment in citrate buffer (pH 6.0; for antigen retrieval), the slides were immersed in 3 % hydrogen peroxide for 20 min to block the activity of endogenous peroxidases. After extensive washing with PBS, the slides were incubated overnight at 4 °C with the anti-LC3 (MBL, Japan) or anti-BNIP3 (Abcam, MA, USA) antibody. The sections were then incubated with a secondary antibody for 1 h at room temperature, and the slides were developed using the STARR TREK Universal HRP detection kit (Biocare Medical, Concord, CA). Finally, the slides were counterstained using hematoxylin. Each slide was imaged at low magnification (×100).

### Biochemistry tests

Whole blood samples from the treated mice were collected by intracardiac puncture and centrifuged at 2000 × g for 20 min to separate the serum. The biochemistry evaluation included assessing the glutamate oxaloacetate transaminase (GOT) activity, glutamate pyruvate transaminase (GPT) activity, albumin levels and blood urea nitrogen (BUN) levels. All experiments and procedures were performed in accordance with the Institutional Care Use Committee guidelines.

### Statistical analysis

Data are expressed as the mean ± SD. Statistical significance was determined using Student’s *t*-test for comparisons between the means or one-way analysis of variance with post-hoc Dunnett’s test [51]. Differences were considered significant when *p* < 0.05.

## Abbreviations

3-MA, 3-methyladenine; AO, acridine orange; ATF6, activating transcription factor 6; AVOs, acidic vesicular organelles; BAF, bafilomycin A1; BNIP3, Bcl-2/adenovirus E1B 19 kDa protein-interacting protein 3; ER, endoplasmic reticulum; IR, ionizing radiation; IRE1α, inositol-requiring enzyme 1α; PERK, PKR-like ER-resistant kinase; UPR, unfolded protein response; UPS, ubiquitin-proteasome system.

## Additional file

Additional file 1: Supplementary results. (DOC 3159 kb)
