# Supplementary material for: Combination of the novel histone deacetylase inhibitor YCW1 and radiation induces autophagic cell death through the downregulation of BNIP3 in triple-negative breast cancer cells in vitro and in an orthotopic mouse model
Source: Mol Cancer. 2016 Jun 10;15:46. doi: 10.1186/s12943-016-0531-5 (PMC4902929; doi:10.1186/s12943-016-0531-5)
Supplement: Additional file 1: — Supplementary results. (DOC 3159 kb) [file 12943_2016_531_MOESM1_ESM.doc]

**Supplementary results**


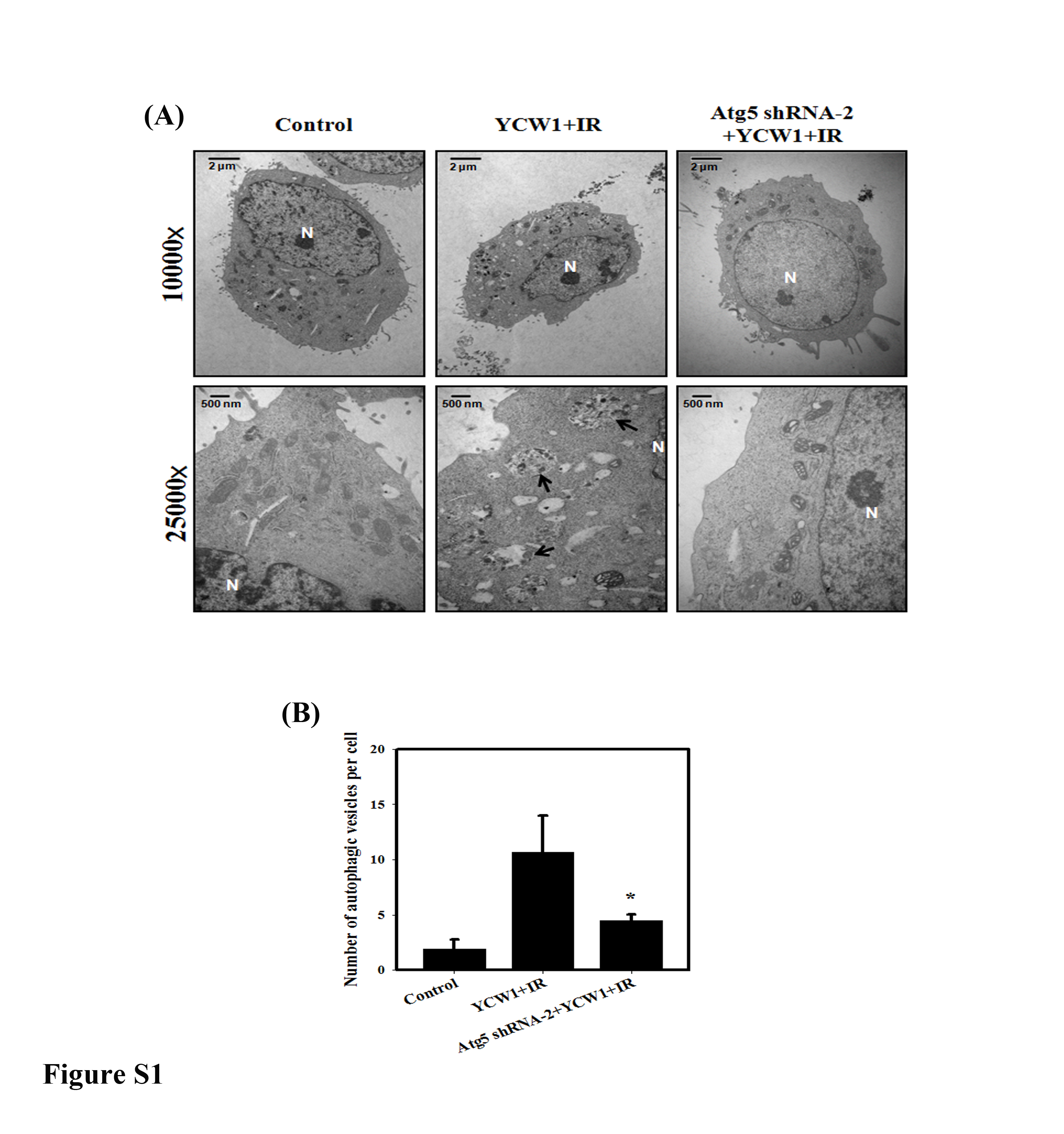


**Figure S1.**

The ultrastructures of 4T1 cells transfected with Atg5 shRNA were analyzed by TEM. (A) TEM photomicrographs in the absence or presence of Atg5 shRNA. N, nucleus. Black arrows, autophagosomes. (B) The number of autophagic vesicles in each cell was determined with 10 cells in each sample, respectively. Cells were transfected with Atg5 shRNA for 24 hrs and were then incubated with YCW1 (1 μM) and IR (4 Gy) for 48 hrs. *, p<0.05, YCW1+IR versus Atg5 shRNA+YCW1+IR.
